# Dietary analysis and patterns of nutritional supplement use in normal and age-related macular disease affected subjects: a prospective cross-sectional study

**DOI:** 10.1186/1475-2891-3-16

**Published:** 2004-09-28

**Authors:** Hannah Bartlett, Frank Eperjesi

**Affiliations:** 1 Neurosciences Research Institute, Aston University, Birmingham, B4 7ET, UK

## Abstract

**Background:**

Poor diet is thought to be a risk factor for many diseases, including age-related macular disease (ARMD), which is the leading cause of blind registration in those aged over 60 years in the developed world. The aims of this study were 1) to evaluate the dietary food intake of three subject groups: participants under the age of 50 years without ARMD (U50), participants over the age of 50 years without ARMD (O50), and participants with ARMD (AMD), and 2) to obtain information on nutritional supplement usage.

**Methods:**

A prospective cross-sectional study designed in a clinical practice setting. Seventy-four participants were divided into three groups: U50; 20 participants aged < 50 years, from 21 to 40 (mean ± SD, 37.7 ± 10.1 years), O50; 27 participants aged > 50 years, from 52 to 77 (62.7 ± 6.8 years), and ARMD; 27 participants aged > 50 years with ARMD, from 55 to 79 (66.0 ± 5.8 years). Participants were issued with a three-day food diary, and were also asked to provide details of any daily nutritional supplements. The diaries were analysed using FoodBase 2000 software. Data were input by one investigator and statistically analysed using Microsoft Excel for Microsoft Windows XP software, employing unpaired t-tests.

**Results:**

Group O50 consumed significantly more vitamin C (t = 3.049, p = 0.005) and significantly more fibre (t = 2.107, p = 0.041) than group U50. Group ARMD consumed significantly more protein (t = 3.487, p = 0.001) and zinc (t = 2.252, p = 0.029) than group O50. The ARMD group consumed the highest percentage of specific ocular health supplements and the U50 group consumed the most multivitamins.

**Conclusions:**

We did not detect a deficiency of any specific nutrient in the diets of those with ARMD compared with age- and gender-matched controls. ARMD patients may be aware of research into use of nutritional supplementation to prevent progression of their condition.

## Background

Poor diet is thought to be a risk factor for many diseases [1,2]. One way of evaluating this risk is to carry out studies using dietary assessment techniques. Food frequency questionnaires (FFQ) have been the primary method of food self-reporting in nutritional epidemiology for the past 20 years, but it is now suggested that the ability to study associations between diet and chronic diseases may be better served by using a food diary [3]. The most accurate methods for dietary assessment are direct observation in the home, or a food history, which involves a 1–2 hour interview by a specially trained nutritionist. These methods are costly and the food diary is often used when they are not possible [4].

Age-related macular degeneration (AMD) is the leading cause of blind registration and visual disability in patients over the age of 60 years in the developed World [5]. The condition affects more than 1.75 million people in the United States, and it is expected that the demographic right-shift will lead to an increase in this number to almost 3 million by 2020 [6]. In accordance with the International Classification and Grading System for Age-Related Maculopathy (ARM), and Age-Related Macular Degeneration (AMD), these abbreviations will be used throughout [7]. The term age-related macular disease (ARMD) will be used to encompass ARM and AMD.

ARM is the early stage of ARMD and is most often clinically apparent over the age of 50 years. The main symptom is increasing difficulty with fine detail discrimination. AMD is the later stage of ARMD and is categorised further in to 'dry AMD' (also known as geographic atrophy, GA), and 'wet AMD' (also known as 'neovascular', 'exudative', or 'disciform' AMD) [7]. GA is the most common form, and is estimated to be present in 15% of eyes by 80 years of age [8-11]. Progression is slow and legal blindness has been estimated to occur between 5 and 10 years [12]. Exudative AMD is less common, occurring in 5.2% of the population over 75 years [13], but accounts for a 90% blind registrations[14]. Patients experience rapid, significant loss of central vision as a result of growth of new blood vessels beneath the retina.

The prevalence of GA and exudative AMD in the US population over 40 years of age has been estimated at 1.47% [95% confidence interval (CI), 1.38% – 1.55%] [6]. The likelihood of visual deterioration in those with exudative AMD may be reduced with laser treatment [15-18], although success is limited. The paucity of treatment options has prompted interest in the identification of risk factors, as well as the development of prevention strategies. The three main risk factors are increasing age [19-26], smoking [22,27-29], and genetic predisposition [30-34], although other proposed factors include gender [35,36], race [37-39], socioeconomic factors [21,40], cardiovascular disease [21,31,41,42], and poor nutrition [43-45].

It is thought that people with low systemic antioxidant levels may be more prone to oxidative damage of the retina and therefore, AMD [46]. Oxidation refers to removal of electrons and is mediated by reactive oxygen intermediates (ROI), which include free radicals, hydrogen peroxide, and singlet oxygen. Free radicals are molecules that contain one or more unpaired electrons in their outer orbits [47], and they extract electrons from other molecules in order to achieve stability. These molecules are rendered unstable by the interaction and a cytotoxic chain reaction results. This damage is thought to contribute to the pathogenesis of many diseases [1,2].

The hypothesised role of oxidation in the development of AMD has prompted research into the use of nutritional supplementation [48]. The Age-Related Eye Disease Study (AREDS) found a significant odds reduction for the development of advanced AMD with antioxidant plus zinc supplementation [49], and the Lutein Antioxidant Supplementation Trial (LAST) reported that visual function in AMD patients is improved with supplementation of lutein and lutein combined with other nutrients [50]. Lutein and its isomer zeaxanthin are carotenoids, and are synthesised in plants, algae, and bacteria. In mammalian systems they can only be obtained from the diet [51]. Their selective absorption by the retina, in particular the macula, is suggestive of a protective function, and has prompted use of the term macular pigment (MP) to describe them within the retina. Lutein and zeaxanthin are believed to protect the retina in two ways. Firstly, they act as blue-light filters. Action spectrum for blue-light induced damage shows a maximum between 400 nm and 450 nm, and this is consistent with the absorption spectrum of macular pigment [52]. Secondly, they are able to quench free radicals. Energy transfer to them quenches singlet oxygen, and they are also believed to react with peroxyl radicals that are involved with lipid peroxidation [53].

The primary aim of this study was to evaluate the dietary food intake of three subject groups: participants under the age of 50 years without ARMD (U50), participants over the age of 50 years without ARMD (O50), and participants with ARMD. The secondary aim was to obtain information on nutritional supplement usage.

## Methods

### Study design

Prospective cross-sectional in a clinical practice setting.

### Participants

Seventy-four participants gave informed consent to take part in this study, which was approved by the Institutional Human Ethics Committee. Recruitment methods included sending information to Birmingham optometrists, ophthalmologists, and a specialist centre for rehabilitation of people with sight loss, an editorial in a local newspaper, recruitment e-mails sent to the Royal National Institute for the Blind (RNIB) and all staff and students at Aston University and Aston Science Park, Birmingham, UK. For analysis the participants were divided into three groups: U50; 20 participants aged < 50 years, from 21 to 40 (mean ± SD, 37.7 ± 10.1 years), O50; 27 participants aged > 50 years, from 52 to 77 (62.7 ± 6.8 years), and ARMD; 27 participants aged > 50 years with age-related macular disease, from 55 to 79 (66.0 ± 5.8 years). All participants were part of a larger study investigating the effects of nutritional supplementation on visual function in normal and diseased eyes [54].

Chi squared analysis for gender yielded no significant difference between groups U50 and O50 [χ^2 ^(1) = 0.104 p = 0.305] and groups O50 and ARMD [χ^2 ^(1) = 3.814 p = 0.051]. The difference in age is not significant between groups O50 and ARMD (t = -1.842, p = 0.071).

Exclusion criteria were the presence of an ocular condition other than ARMD and the presence of medical conditions indicating a diet in which particular foods or food groups were excluded (e.g. coeliac disease).

Participants were issued with a three-day food diary with verbal and written instructions explaining that they should add to their diary every time they eat or drink, describing the food as accurately as possible and giving estimates of amounts. They were also asked to provide details of any daily nutritional supplements. The diary consisted of two week days and one weekend day. The diaries were analysed using FoodBase 2000 software (The Institute of Brain Chemistry and Human Nutrition, 166–220 Holloway Road, London N7 8DB, UK), which is a computerised nutrition database containing data on approximately 3750 foods. It can be used for recipe analysis, meal analysis, and daily or weekly analysis of menus or food intakes. Data were input by one investigator and statistically analysed using Microsoft Excel for Microsoft Windows XP software, employing unpaired t-tests.

## Results

### Dietary analysis

The values for energy and nutrient intakes for all participants are shown in table 1.

**Table 1:** Daily mean and SD values for energy and nutrient intake.

	Group U50 (mean ± SD) n = 20	Group O50 (mean ± SD) n = 27	Group ARMD (mean ± SD) n = 27
Energy (kcals)	1672.30 ± 425.58	1599.78 ± 331.50	1823.37 ± 546.18
Protein (g)	71.91 ± 27.62	68.14 ± 17.08	85.25 ± 18.28
Fat (g)	65.82 ± 27.77	58.44 ± 44.27	66.73 ± 21.76
Carbohydrate (g)	203.13 ± 39.46	204.43 ± 44.27	222.05 ± 82.66
Alcohol (g)	4.31 ± 4.38	4.83 ± 8.70	6.86 ± 9.54
Fibre (g)	13.04 ± 4.60	16.21 ± 5.44	17.31 ± 5.47
Cholesterol (mg)	192.19 ± 122.99	203.04 ± 82.63	242.60 ± 72.46
Zinc (mg)	8.43 ± 2.98	8.50 ± 2.58	10.07 ± 2.45
Copper (mg)	1.10 ± 0.52	1.21 ± 0.51	1.43 ± 0.54
Selenium (μg)	62.82 ± 75.84	105.01 ± 126.05	72.01 ± 62.76
Riboflavin (mg)	9.90 ± 35.82	1.57 ± 0.43	1.77 ± 0.44
Vitamin C (mg)	79.01 ± 40.67	140.59 ± 75.23	114.91 ± 60.01
Vitamin E (mg)	6.33 ± 3.49	6.71 ± 3.13	7.87 ± 3.52
Vitamin D (μg)	3.33 ± 3.06	3.05 ± 1.91	3.76 ± 2.54
Retinol equivalents (μg)	681.25 ± 499.55	679.44 ± 237.65	825.22 ± 440.54
% energy from fat	34.20 ± 7.34	32.56 ± 4.69	32.89 ± 5.60

#### Comparing group U50 with group O50

Group O50 consumed significantly more vitamin C (t = 3.049, p = 0.005) and significantly more fibre (t = 2.107, p = 0.041) than group U50.

The mean intakes for men and women in each group are shown in table 2. The data has been broken down into male/female subgroups because reference nutrient intake (RNI) data can differ with gender.

**Table 2:** Mean vitamin C and fibre daily intake for groups U50 and O50

	Mean vitamin C intake (mg)	Mean fibre intake (g)
Women under 50 years (n = 12)	77.48 ± 42.79	12.19 ± 4.06
Women over 50 years (n = 22)	147.13 ± 75.12	16.03 ± 4.92
Men under 50 years (n = 12)	78.68 ± 36.93	14.18 ± 4.78
Men over 50 years (n = 7)	125.87 ± 78.20	15.96 ± 6.48

By tradition, investigators consider a study to be adequately powered if it has an 80% chance of detecting a significant difference when one exists. The number of study participants needed to detect a clinically important difference with acceptable power, can be calculated using the required power, the expected variability of the outcomes, and the chosen probability of masking a type 1 error [55]. Power analysis shows that 20 subjects is not sufficient to have an 80% chance of detecting a difference of 25% or more of the mean value at the 5% level of significance using the unpaired t-test for alcohol, copper, cholesterol, selenium, vitamin E, vitamin D, and retinol equivalents. In other words, for these dietary consitiuents we cannot state whether we found no difference between groups because there actually was no difference, or because the study did not have enough power to detect a difference. The study was however, powered to assess the difference in means for energy, protein, fat, carbohydrate, zinc, riboflavin, and percentage energy from fat, and no significant differences were found.

#### Comparing group O50 with group ARMD

Group ARMD consumed significantly more protein (t = 3.487, p = 0.001) and zinc (t = 2.252, p = 0.029) than group O50 (see table 3). Power analysis shows that 27 subjects is not sufficient to have 80% chance of detecting a difference in means of 25% at the 5% level of significance using the unpaired t-test for alcohol, copper, cholesterol, selenium, vitamin E, vitamin D, and retinol equivalents. The study was powered to assess the difference in means for energy, fat, carbohydrate, fibre, riboflavin, and percentage energy from fat, and found no significant difference.

**Table 3:** Mean zinc and protein daily intake for groups O50 and AMD

	Mean zinc intake (mg)	Mean protein intake (g)
Women over 50 years (n = 22)	8.30 ± 2.50	66.35 ± 16.12
Men over 50 years (n = 7)	9.25 ± 2.50	71.61 ± 17.46
Women with ARMD (n = 13)	9.60 ± 2.26	78.05 ± 15.98
Men with ARMD (n = 14)	10.51 ± 2.54	91.93 ± 17.73

### Baseline nutritional supplement intake

**Figure 1:**
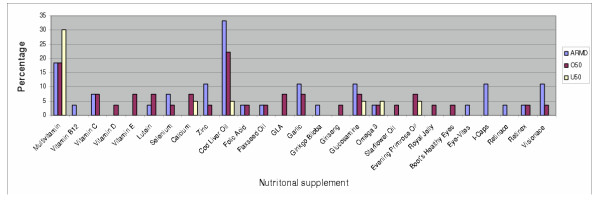
Daily nutritional supplement use by group.

The results indicate that group O50 (mean ± SD; 1.44 ± 1.79) consumes significantly more types of nutritional supplement than group U50 (0.55 ± 1.11) (t = 2.220, p = 0.032). No difference found between groups O50 and ARMD, and the study was powered to have an 80% chance of detecting a difference in means of 1 at the 10% level of significance. The percentage of supplements taken for each group is shown in figure 1.

## Discussion

The aim of this study was to evaluate the dietary intakes of three subject groups; U50, O50 and ARMD, as well as to obtain information on nutritional supplement usage. Participants under the age of 50 years consumed significantly less dietary vitamin C than those aged over 50 years. Supplementation data shows that 7.4 % of the O50 group take uncombined vitamin C compared with 0% of the U50 group. However, a higher percentage of the U50 group take multivitamins (33.3%) compared with the O50 group (22.2%).

Vitamin C is water-soluble, is involved with several biological processes. As a reducing agent it is thought to be active in protection against heart disease. It protects LDL (low density lipoprotein) cholesterol from oxidative damage and reduces platelet aggregation [56]. By enhancing nitric oxide activity, vitamin C is potentially important in lowering blood pressure [57].

High dose supplementation with an antioxidant and zinc formulation, including vitamin C was associated with a 25% reduced risk of progression of AMD in those participants already suffering with the condition [49]. Some studies, however, have found no evidence for a beneficial role of vitamin C supplementation in ocular disease. There was no relationship between cataract prevalence and vitamin C intake in two studies [58,59], and no relationship between cataract extraction and vitamin C intake in a third [60].

Although the antioxidant properties of vitamin C are well known, there is no clinical evidence suggesting that supplementation with vitamin C can reduce the risk of ARMD, or other ocular conditions such as cataract and glaucoma. The RNI for men and women over the age of 18 years is 40 mg. The mean intakes of men and women in the U50 and O50 groups are all above this value (table 2). The higher intake values of the O50 group may be explained by their increased awareness of the benefits of a balanced diet, consumption by this group of more traditional, home prepared foods, and lower consumption of convenience foods. An increased consumption of convenience foods in the U50 group may also explain why they consume significantly less fibre than the O50 group (t = 2.107, p = 0.041). Interestingly, all three groups had a mean intake value of less than 18 g, the RNI for fibre in men and women.

The ARMD group consumed significantly more dietary zinc than age- and gender- matched controls. Zinc has been investigated with regard to its potential preventative role in ARMD. The AREDS group found a suggestive reduction in the risk of progression of AMD in participants supplementing with 80 mg zinc daily. Previous randomized controlled trials (RCTs) using 200 mg zinc daily found conflicting results [61,62], and the positive result reported by Newsome *et al *(1988) should be treated with caution [48]. The higher intake by ARMD participants may be explained by their awareness of research into zinc supplementation and the condition. The RNI for women over 18 years is 7.0 mg and for men over 18 years is 9.5 mg. Our results show that the mean intakes were above RNI values for all four groups. Supplementation data shows that 11.1 % of the ARMD participants supplemented with zinc, compared with 3.7 % of the O50 group, and 0 % of U50 participants. The Food Standards Agency released a report on the safety of vitamins and minerals in May 2003 and suggested a safe upper limit of 42 mg for total daily zinc intake. Zinc supplementation over 150 mg/day has been associated with gastrointestinal side effects such as cramping and nausea, as well as lethargy and blood in the urine [63]. Our results show that the ARMD participants are most at risk of exceeding the safe upper limit as they have the highest dietary and supplemental zinc intake.

The ARMD group consumed significantly more protein than O50 participants. We are not aware of any investigation into a link between protein and risk of ARMD, and table 4 shows that the mean intakes are above the RNI for both men (55.5 g) and women (46.5 g).

Previous studies have found a relationship between higher dietary fat intake and risk of ARM (RR 1.6) [64], and high serum cholesterol and increased risk of exudative AMD compared with low serum cholesterol levels [relative risk (RR) 4.1] [40,65]. However, the NHANES I found that subjects with high cholesterol intake were less likely to develop AMD than those with lower intake [odds ratio (OR) 5.1] [21]. Our results show that ARMD participants consumed more fat and cholesterol than the O50 group, although these differences were not statistically significant. The study was underpowered for cholesterol.

Research into the role of alcohol consumption in the development of AMD has produced conflicting results. Several studies have found no relation [40,66-70], but consumption of beer has been related to an increased risk of retinal pigmentation (OR 1.13) and exudative AMD (OR 1.41) [71]. Both men (RR 2.16) and women (RR 2.20) in the highest category of wine intake (2 or more glasses per day) have been shown to be at increased risk of AMD [67]. This association was strongest with white wine, and interestingly the NHANES I determined that red wine is associated with a lower risk of AMD [21]. This may be related to the antioxidant properties of the phenolic compounds within red wine [72]. Our data shows that the ARMD group consumed more alcohol than both the U50 and O50 groups, although these differences were not statistically significant and the study was underpowered for alcohol.

The non-significant differences found between groups for alcohol, copper, cholesterol, selenium, vitamin E, vitamin D, and retinol equivalents may have occurred because there truly was no difference, or because the study had insufficient power to detect a difference. Because of the variability of the data, subject numbers required per group for 80 % power at the 5 % significance level are 467 for alcohol, 50 for copper, 44 for cholesterol, 341 for selenium, 59 for vitamin E, 113 for vitamin D, and 71 for retinol equivalents.

Multivitamins were the most commonly taken supplement by the U50 group (30.0 %), compared with cod liver oil for the both the ARMD group (33.3 %) and O50 participants (22.2 %). Seventy-five percent of the specific ocular health related supplements were taken by the ARMD group, 25 % by the O50 group, and 0 % by the U50 group.

## Conclusion

We did not detect a deficiency of any specific nutrient in the diets of those with ARMD compared with age- and gender-matched controls. A higher percentage of ARMD participants consume specific ocular health nutritional supplements (33.3 %) compared with age- and gender-matched controls (11.1 %) and U50 participants (0 %). The U50 group consumed a higher percentage of multivitamins, but significantly less vitamin C and fibre than the O50 group. This suggests that the younger age-group might use supplementation to ensure adequate consumption of vitamins and minerals. The ARMD group consumed more dietary zinc, more supplemental zinc, and the highest percentage of ocular health related supplements. This may suggest that information regarding the results of studies investigating the role of nutritional supplementation in reducing the risk of onset or progression of AMD is reaching patients. These results however, may be confounded by the fact that the ARMD participants used in this study were enrolled in an RCT investigating the use of nutritional supplementation in ARMD. Participants in research projects may be more aware of scientific developments and more likely to investigate their condition and potential therapies.

## Competing interests

The authors declare that they have no competing interests.

## Authors' contributions

HB participated in the design of the study, carried out data collection, input, and analysis, and drafted and developed the manuscript. FE participated in the design of the study and development of the manuscript. Both authors read and approved the final manuscript.
